# Orthodontic Treatment Timing and Modalities in Anterior Open Bite: Case Series Study

**DOI:** 10.2174/1874210601711010581

**Published:** 2017-11-16

**Authors:** Wisam Al Hamadi, Fayez Saleh, Mohamad Kaddouha

**Affiliations:** 1 University of Babylon, College of Dentistry, Hillah, Iraq; 2 Department of Orthodontics, Beirut Arab University, Beirut, Lebanon; 3 Private practice, Beirut, Lebanon

**Keywords:** Anterior open bite, Early treatment, Adult treatment, Surgical correction, Habit breaker, Fixed palatal crib

## Abstract

**Objective:**

The purpose of this study was to present early and adult cases of anterior open bite that were treated efficiently using different treatment approaches and mechanics.

**Materials and Methods:**

Five patients of different age groups (from 7 to 27 years), suffering from a clear Anterior open bite deformity, were properly diagnosed and relevant treatment modality for each was selected.

**Results:**

Positive overbite was efficiently achieved for all patients.

**Conclusion:**

Patient compliance is a key factor in using removable habit breakers. However, fixed palatal crib gave the same results but in shorter time. Anterior open bite of skeletal components should be thoroughly evaluated before selecting camouflage or orthognathic surgery treatment modality.

## 1 INTRODUCTION

Early orthodontic treatment is becoming more generally accepted as a means of gaining the greatest possible control over mal-growing dentofacial components including anterior open bite. However, in most cases, a second phase of treatment may be necessary to detail the occlusion and maintain life-long occlusal stability; while in others camouflage or even orthognathic surgery is recommended if standard outcome of facial esthetics and oral function are to be achieved.

Speidel *et al*. [1] in a review of the etiology, diagnosis and treatment of anterior open bite, stated that due to the complexity of etiologic factors of anterior open bite, each individual case requires careful and thorough examination to formulate a proper diagnosis and treatment plan for that patient. In a recent study on Italian preschool children, Silvestrini-Biavati *et al*. [2] pointed out the fact that non-nutritive sucking habits are essential etiologic factor of developing anterior open bite in deciduous dentition in addition to type of feeding. Gianelly [3] concluded that 90% of all growing patients can be treated successfully in one-phase protocol if treatment was started in the late mixed dentition. However, the pause between phase I and phase II encountered certain degree of relapse and this led to long-term retention protocol.

Pinkham *et al*. [4] studied the atypical swallowing habits and the role of the tongue malfunction or tongue–thrusting habit in causing disruption of equilibrium of forces between oral and perioral musculature. They concluded that this imbalance may impede eruption of individual or segments of the dentition and consequently causes anterior open bite. However, Proffit and Mason [5] considered that tongue-thrust habit may be able to sustain open bite but not create it.

McNamara and Brudon [6], and Subtelny [7] among others reported that if etiologic factors could be recognized and treated early, then it might be possible to minimize or even eliminate most of the developing dentoalveolar deformities.

Maciel and Leite [8] in a review on the etiologic aspects of anterior open bite and their implications to the oral functions reiterated that the therapeutic success of anterior open bite depends on the close collaboration with the otorhinolaryngologist and allergist to eliminate any upper airway respirator obstruction, in addition to other myofunctional and occlusal problems.

In another study, NG *et al*. [9], addressed the major challenge a clinician encounters when treating anterior open bite which is the patient’s concern about oral function and facial esthetics. They have discussed the non-surgical treatment options of anterior open bite (preventive, interceptive and camouflage) and pointed out to the fact that orthognathic surgery may be the only effective and stable approach left for adult skeletal open bite.

Ballanti *et al*. [10] evaluated the Dentoskeletal features of subjects with AOB in the mixed dentition using both conventional cephalometric analysis and morphometric analysis applied to PA films. Their results exhibited significant shape differences in craniofacial configuration mainly as transverse contraction of the zygomatic region of the maxilla (both skeletal and dentoalveolar) and of the mandible at the condylar and gonial levels. The mandible also showed tendency toward vertical elongation (hyperdivergent) in AOB subjects when compared to control group of normal overbite.

Ribeiro *et al*. [11] reported a successful non-surgical treatment for an adolescent female presented with Angle Class III malocclusion, excessive lower facial height, and anterior open bite. The patient refused the orthosurgical treatment modality and another option was suggested using multiloop edgewise archwire (MEAW) in association with a chincup to correct the divergence of occlusal planes, molar relationship, without major change of the patient’s profile.

Zuroff *et al*. [12] evaluated the post-treatment stability of orthodontic open bite correction after 10 years of retention. Subjects were treated with different nonsurgical modalities. They concluded that it was not possible to predict which patients would yield more stable results when using their pretreatment records.

Gracco *et al*. [13] re-treated a relapsed adult case of anterior open bite non-surgically by resolving the main causative factor that caused the relapse. Head cone-beam computed tomography revealed nasal airway breathing problems due to nasal septum deviation, turbinate hypertrophy, and maxillary sinus congestion. They emphasized on the pretreatment consultation of otorhinolaryngologist prior to considering the treatment of anterior open bite.

Leite *et al*. [14] found that the palatal crib and bonded spurs are beneficial in breaking sucking habits and in maintaining the tongue posture, thus preventing the development of anterior open bite in growing patients. However, fixed palatal crib is more beneficial than removable appliances or bonded lingual spurs as it increases the stability of the dentofacial morphologic correction.

A Cephalometric study conducted on 107 school children (77 participants + 30 as control group) by Insabralde *et al*. [15] elucidated the dentoskeletal effect of removable palatal crib, bonded spurs, and chincup therapy on anterior open bite developed in growing children. They concluded that removable palatal crib and bonded spurs were responsible for dental AOB correction; chincup controlled the vertical development of maxillary molars (intrusion) and relatively reduced the long vertical facial height without significant effect on the dento-alveolar components of AOB. Therefore, chincup should not be used alone but with other appliances to take full benefit of the growing stage skeletally and dentally.

The purpose of this clinical study was to present early and adult cases of anterior open bite which were treated efficiently using different treatment approaches and mechanics.

## 2 CASE REPORT

### 2.1 Case # 1

A young male aged 8 years 7 months presented with the following dentofacial problems:

Lip Biting Habit → Open bite, incompetent Lips,

Tongue Thrust, ↑ Overjet, Median Maxillary Diastema,

Tongue Pressure Prevents Maxillary Laterals from Eruption.

The parents’ and patient’s chief complaint was the anterior open bite and facial disharmony.

His medical history was good and free from any relevant health problems.

In view of the pretreatment records and clinical examination, interceptive orthodontics treatment was recommended using removable Hawley appliance with screening device and habit breaker as shown in Fig. (**1**).

**Fig. (2):**
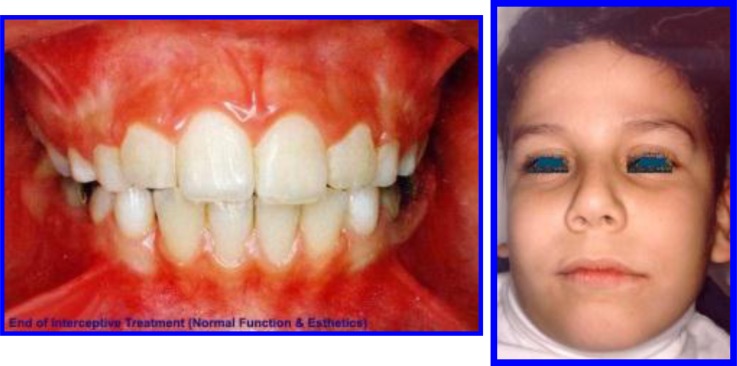
Post phase 1 treatment after 16 months duration, normal development of occlusion in 3Ds was achieved. The extra-oral features were in harmony and balance (competent lips and pleasant facial components). Case # 1.

Phase I early treatment was successful and further follow up confirmed parents’ and patient’s satisfaction. A retention protocol was established to maintain future stability (Fig. **2**).

### 2.2 Case # 2

A female aged 8 years, 2 months, presented with Class I malocclusion and pleasant facial features.

**Fig. (3):**
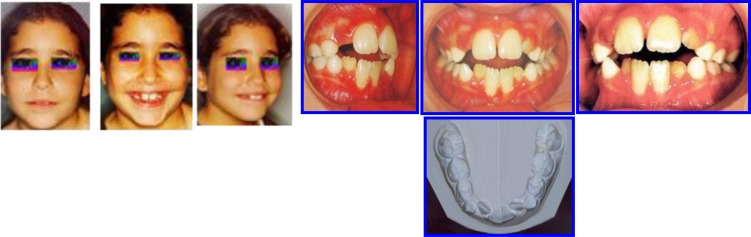
Pre-treatment records: Extra-oral and Intra-oral features. Case # 2.

**Extra-oral examination:** The patient had a rounded, symmetric face with incompetent lips where the lower lip is tucked behind the maxillary incisors forcing them labially and the mandibular incisors lingually (Fig. **3**). Her past medical history was free from any health problem.

**Fig. (4):**
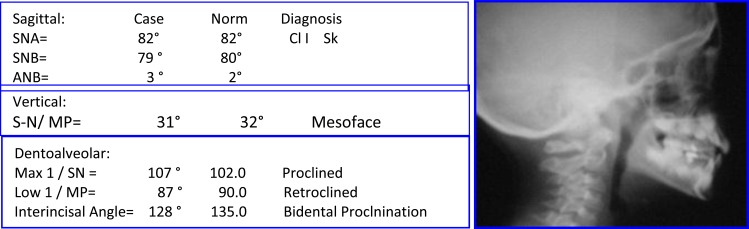
Pretreatment Cephalometric analysis of skeletal and dento-alveolar components Case # 2.

**Intra-oral examination revealed the following features (Fig. **4**).**

- Anterior open bite with normal Cl I Occlusion
- Increased Overjet (Max Incisors Protruded)
- Tongue thrust & atypical swallowing pattern
- Lower lip entrapped under upper lip at rest
- Lower anterior arch crowding
- Lower anterior gingival recession
- Oro-nasal breather
- Distorted occlusal plane

**Fig. (5):**
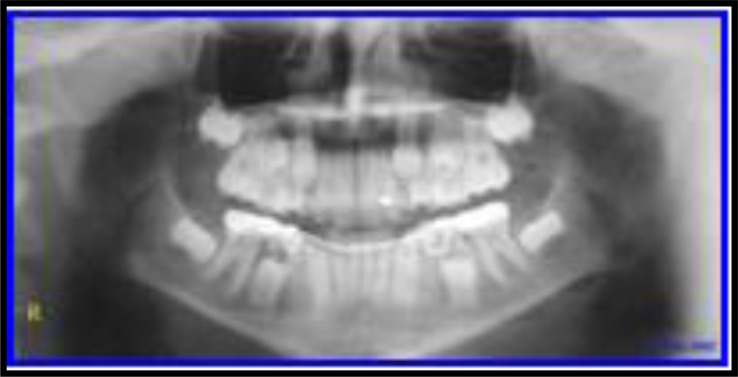
Pre-Ortho treatment panoramic view. case# 2.

**Panoramic Radiograph (Fig. **5**) showed:**

-Early Mixed Dentition Phase with normally developing dentition-
-Lower anteriors + 4 1st molars erupted and roots are completed,
-Max Right Lateral did not erupt yet
-Max left lateral is partially erupting.
-All other teeth are present except third molars.

The patient's chief complaint was a lack of incising ability with anterior teeth and abnormal lip seal.

**Fig. (6):**
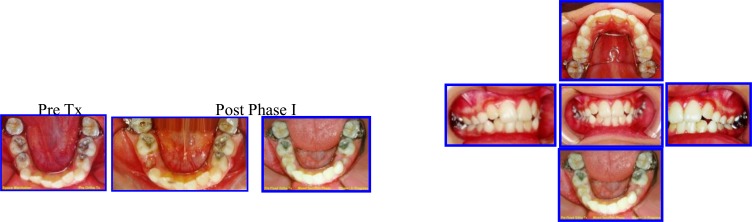
Initial interceptive orthodontic appliances in both arches and treatment progress. Case # 2.

**Fig. (7):**
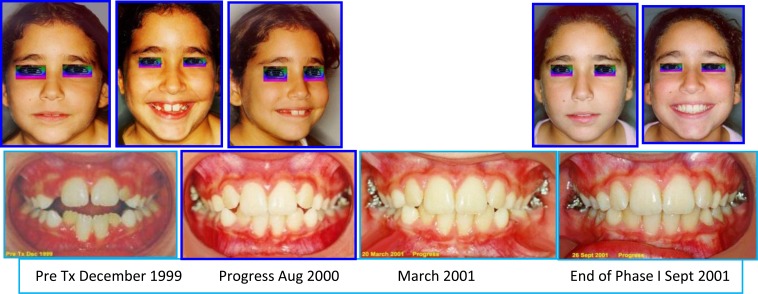
Facial & occlusal features (pretreatment, early & end of phase I). Case #2.

**Fig. (8):**
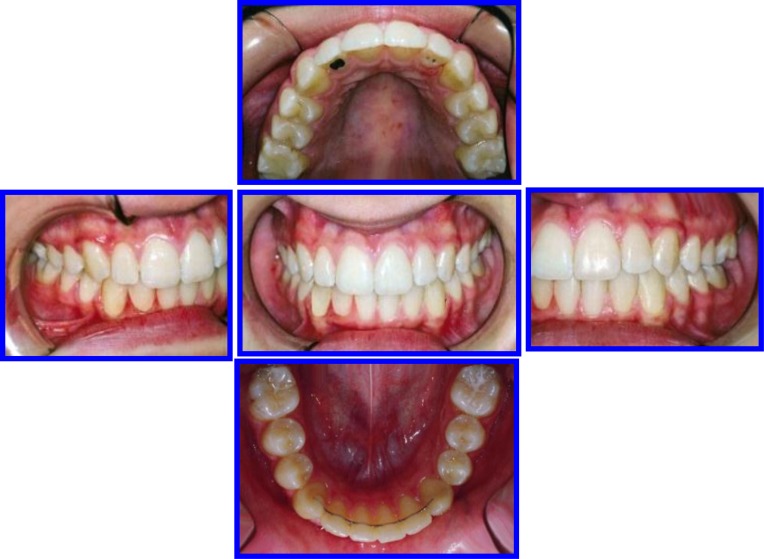
End of Phase II treatment showing radiographs, facial & occlusal features. Case #2.

-Early Orthodontic Treatment (Lingual Arch) Started at 21 Oct 1999- (Phase I) (Figs. **6**-**8**)

- Start of Fixed Tongue Crib + TPA (Goshgarian)
- Ant Teeth Eruption & Crowding Resolved (Nov 2000)
- Early Treatment Finished at 22 July 2002
- 1st Phase Treatment Duration 2 Ys + Lower Arch Development

### 2.3 Case # 3

**Fig. (9):**
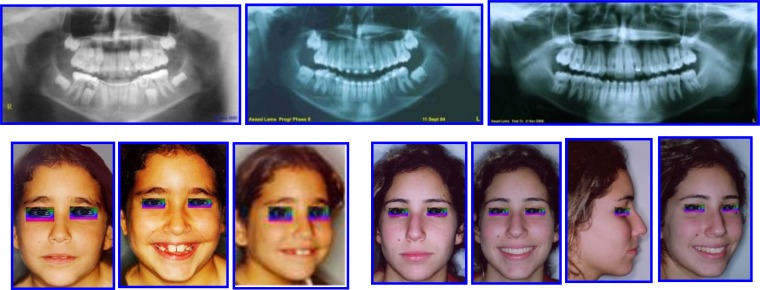
Pretreatment records: Facial & occlusal features. Radiographs: Ceph analysis & Panoramic. Case # 3.

Diphasic Treatment of AOB & Narrow Maxilla (Fig. **9**).

A female Aged 7y 5M, presented with pleasant face, AOB, incompetent lips, gummy smile, tongue thrust, narrow maxilla and *convex mild dolichoface.* Her medical history is free from any health problems.

**Fig. (10):**
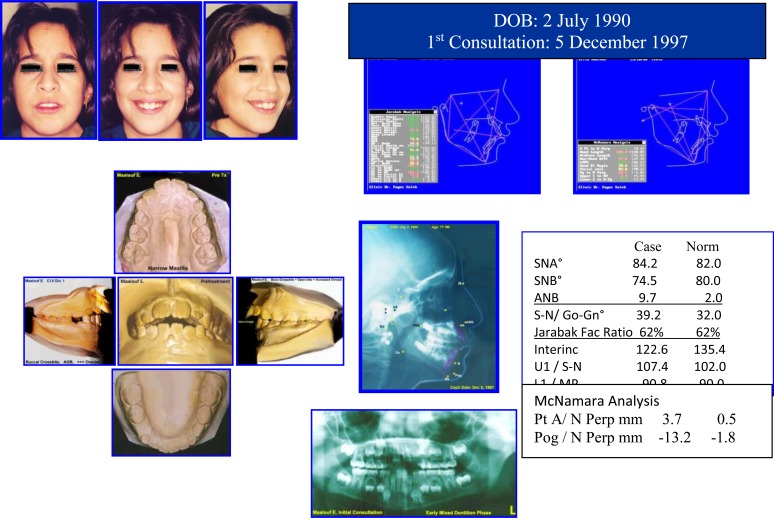
Sequence of Phase I treatment: RPE (Haas Appliance); Followed by hawley retainer with tongue guard for stability (retention), ready to receive phase II treatment. P.6, case # 3.

**Fig. (11):**
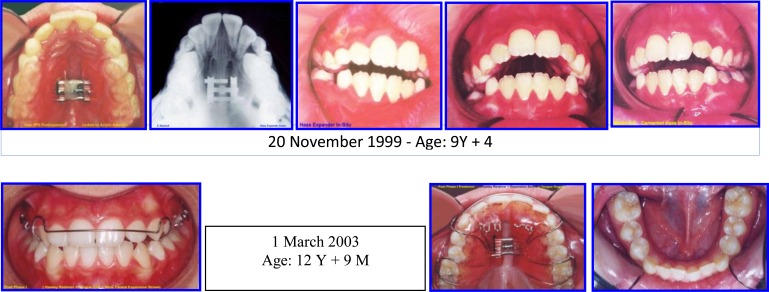
Phase II (Comprehensive Ortho Treatment) - June 2003-October 2004 Pre-finishing Stage. Case # 3.

**Fig. (12):**
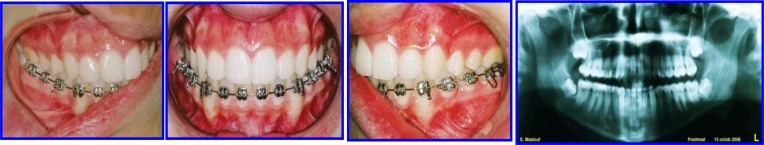
Post-treatment facial & occlusal features. Case # 3.

Sequence of Phase I Treatment: RPE (Haas Appliance); Followed by Hawley Retainer with Tongue Guard for Stability (retention), ready to receive Phase II Treatment (Figs. **10**-**12**).

**Fig. (13):**
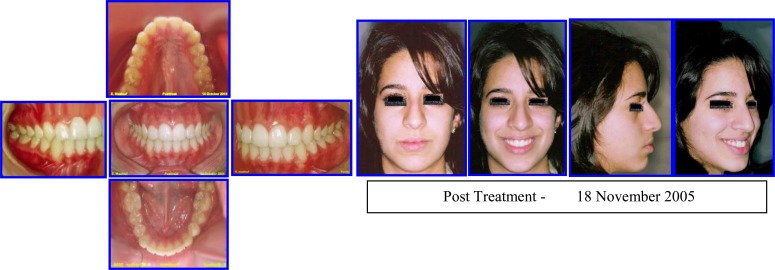
Three years post retention facial & occlusal features (October 2008). Case # 3.

After three years post retention facial & occlusal features (October 2008) (Fig. **13**).

### 2.4 Case # 4

**Fig. (14):**
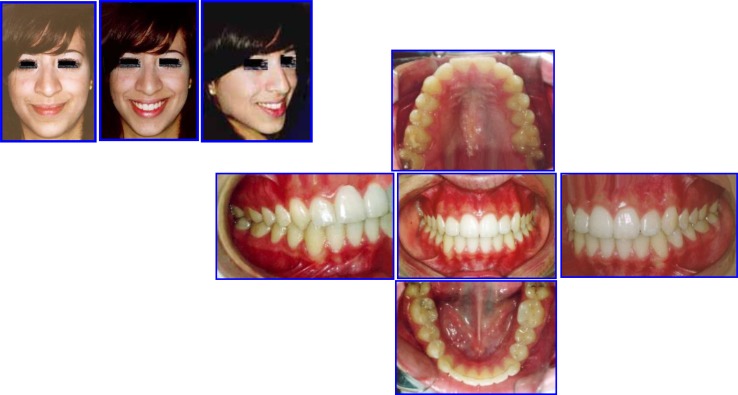
Pretreatment records: Facial & occlusal features. Ceph tracing & analysis. Case # 4.

**Fig. (15):**
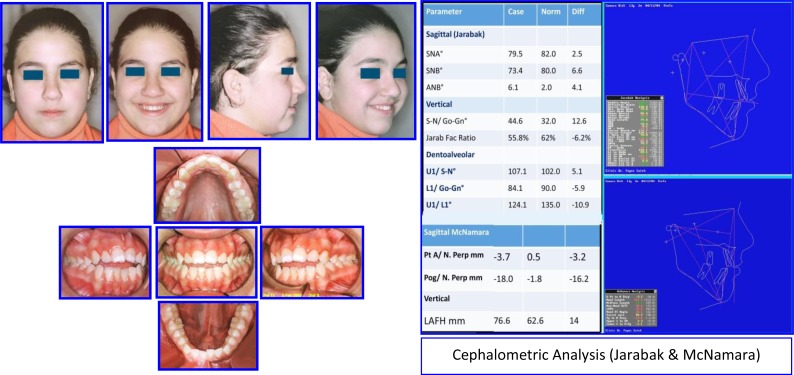
Sequence of comprehensive ortho treatment, from habit breaker to detailing of occlusion. Case # 4.

**Fig. (16):**
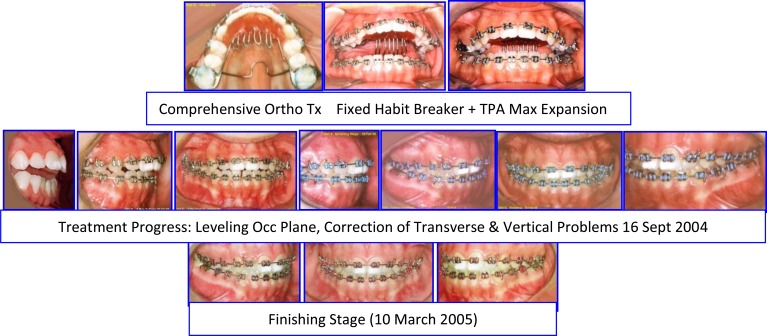
Post Tx: Occlusion & facial esthetics (19 April 2007). Case # 4.

Female: (Age 13 Y + 2 M) 2004, Early Adult Orthodontic Treatment of Anterior Open Bite Cephalometric Analysis (Jarabak & McNamara) (Figs. **14**-**16**).

**Problem Listing:** Records: 5 February 2005

- Anterior Open bite - Distorted Occ Plane
- Mild Crowding - Buccal Crossbite
- Tendency Dolichoface - Pleasant Facial Esthetics

**Etiology**

- Tongue Thrust Habit
- Mild Oro-Nasal Breathing? Resolved Prior to Treatment

### 2.5 Case # 5

Orthosurgical Correction of Anterior Open Bite

Adult Female 27Y old with sequence of comprehensive ortho treatment of occlusion.

**Fig. (17):**
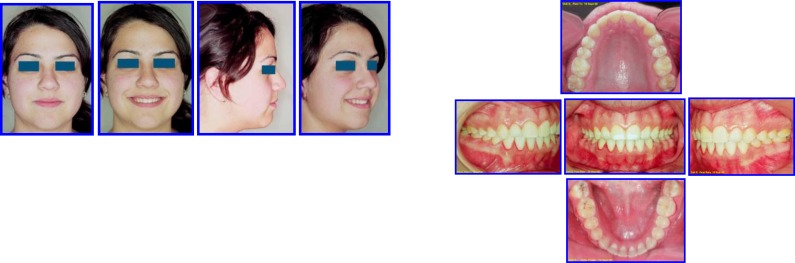
Pre-treatment records: Facial & occlusal features. Case # 5.

**Fig. (18):**
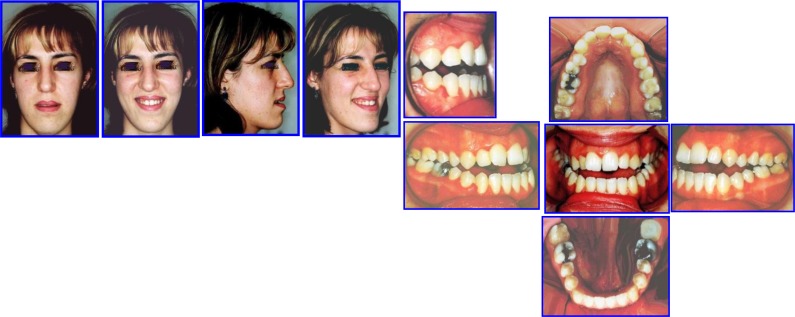
Radiographic examination: Panoramic & cephalometric analysis. Case # 5.

**Major Extra-Oral Findings** (Figs. **17**, **18**).

- Long Face Syndrome
- Gummay Smile
- Anterior Open Bite
- Class III Malocclusion
- Mandibular dental deviation to the left 4mm
- Incompetent Lips
- Narrow alar bases

**Intra-Oral Examination**

- Complex open bite (5mm)
- Bilateral Buccal Crossbite
- Distorted Occlusal Plane
- Midline Deviation
- Class III Malocclusion (Right full unit; left half unit)
- Moderate Max Crowding & minimal crowding in the lower arch
- Zero Overjet

**Fig. (19):**
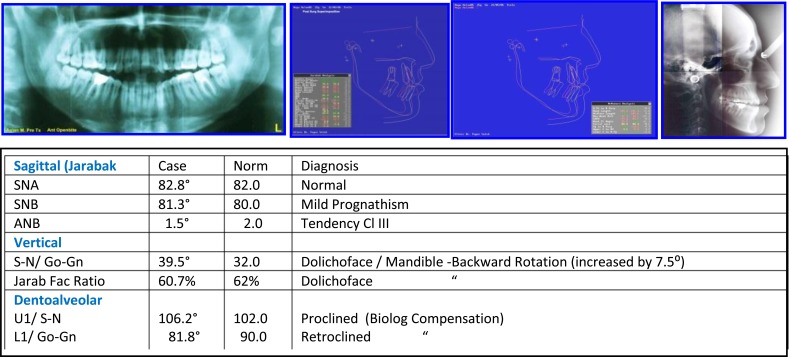
Pre-surgical orthodontic preparation/ intra-arch levelling & alignment / Occ Plane. Case # 5.

In addition to radiographic examination (Fig. **19**).

**Fig. (20):**
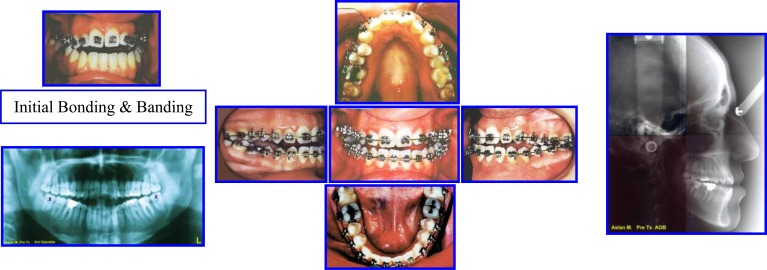
Surgical procedures and post-surgical stabilizing of the occlusion in 3 Ds. Case # 5.

**Fig. (21):**
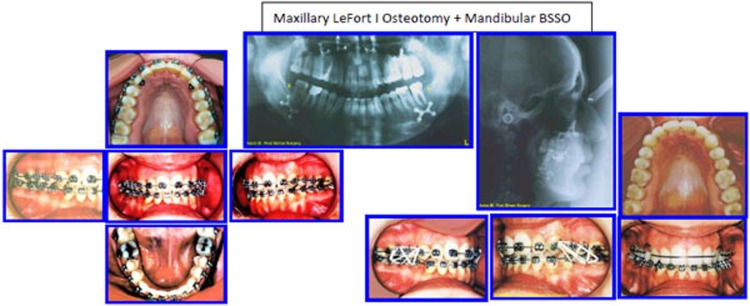
Soft and dentoskeletal tissues improvement post-surgical orthodontics. Case # 5.

**Fig. (22):**
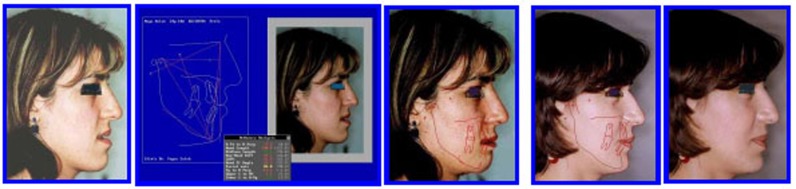
Pre treatment profile, soft tissue prediction/ surgical planning and post surgery for case # 5.

**Fig. (23):**
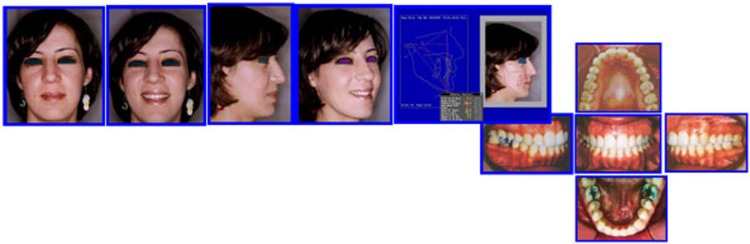
Post treatment, facial esthetics & occlusionfor case # 5.

**Fig. (24):**
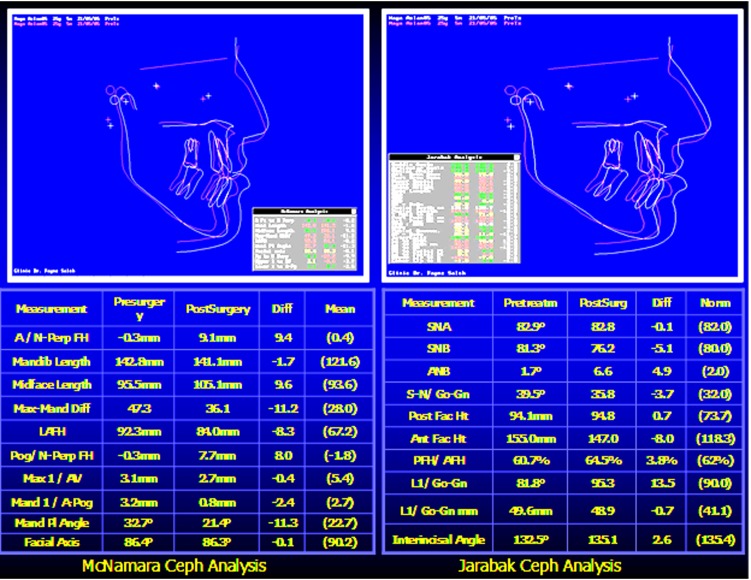
Cephalometric superimposition (McNamara & Jarabak analyses) showing the post-operative improvement of dentofacial features for Case # 5.

The patient was treated with presurgical alignment and postsurgical treatment and evaluated with cephalometric analysis to improve the treatment (Figs. **20**-**24**).

#### Treatment Objectives and Planning

The skeletal and dentoalveolar complexities of the case necessitated a combined consultation and treatment planning with the maxillofacial surgeon. To restore normal facial esthetics and occlusion; the following schedule was agreed upon:

1. Presurgical comprehensive orthodontic treatment including removal of the lower wisdom teeth;
2. LeFort 1 osteotomy with superior positioning of the maxilla and bilateral sagittal split osteotomy of the mandible to restore normal lip-teeth relationship and reduce the increased facial height; and
3. Postsurgical orthodontic care to settle the occlusion and maintain future stability of the treatment outcome.

## 3 DISCUSSION

Each individual case presented in this study required a careful and thorough clinical examination to formulate a proper diagnosis and treatment plan. The first three cases were in the growing age and therefore, early correction of anterior open bite was easier because it is mainly dental in nature aided by greater growth potential of the dental arches (Spidel *et al*. 1972) [1]. Eliminating any upper airway respiratory obstruction in the late mixed dentition stage also helps to control myofunctional and occlusal problems (Maciel and Leite, 2005) [8]. Monophasic or diphasic early orthodontic treatment depends on the complexity of the deformity where a range of nonsurgical treatment modalities exist as reviewed by NG *et al*. (2007) [9]. **Case # 1** was resolved in one phase early treatment, while the other two cases necessitate a diphasic treatment modality; phase I to resolve the dentoalveolar deformity anterior open bite and phase II to correct any remaining intra and inter-arch discrepancy (Gianelly, 1995) [3] and (Leite *et al*. 2015) [14]. The cause and effect debate between tongue–thrusting habit and anterior open bite although not yet resolved, from a therapist point of view, yet early intervention using a simple and efficient appliance (as in case # 1) succeeded in promoting normal growth pattern, correction of the dentofacial deformity and avoidance of relapse, this was confirmed by Insabralde *et al.* (2016) [5]. **Cases # 2 and 3** were planned for diphasic orthodontic treatment to take the advantage of turnover of the growing tissues followed by a pause interval retaining the arch forms and giving chance for the eruption of teeth and their roots formation. Comprehensive orthodontic phase II treatment detailed the occlusion and overcorrected the AOB. **Case # 4**, an early adult female with all teeth erupted except third molars. Data collection revealed a well-balanced face with a tendency to increase lower anterior facial height, AOB, mild crowding, distorted occlusal planes, tongue thrust habit, and oro-nasal breathing which was resolved prior to treatment as advised by Gracco *et al*. (2015) [13], A fixed palatal tongue crib with TPA was constructed to relocate the tongue and expand the buccal segment as recommended by Ballanti *et al*. (2009) [10]. Leveling arch wires were inserted to level the occlusal plane. Vertical elastics helped to relate properly the arches and stabilize the occlusion, positive overcorrected overbite was achieved and esthetic anterior occlusal plane restored the normal lip-teeth relationship and esthetic smile, according to Zuroff *et al*. (2010) [12] positive overbite, breaking the oral habits, and ensured retaining of the treatment outcome are essential to alleviate the concern about future relapse. **Case. # 5 (**adult female 27 years old), was assigned for combined bimaxillary orthosurgery after thorough examination of the pretreatment records by the health care team. Problem listing revealed a complex open bite, gummy smile, class III malocclusion, long face syndrome, and midline deviation. A realistic treatment planning was formulated aiming to achieve stable, functional, and esthetic results. Presurgical preparation corrected the biologic compensation of occlusal relationship and leveled the occlusal planes to facilitate the mobilization of maxillae into normal skeletal and dental relationship in 3 dimensions, the vertical facial excess was reduced by 8mm and anti-clockwise mandibular rotation by 11.3^0^; in the sagittal direction, the SNB angle was reduced by 5.3^0^ and pogonion point retruded 8mm. The p patient has reported a better self-esteem and satisfaction with the dramatic skeletal, dental, and occlusal improvement. Abraham *et al.* (2012) [16] in a similar clinical study emphasized the importance of collaboration of orthodontist, maxillofacial surgeon, and other disciplines to determine the success of orthosurgical outcome beforehand.

## CONCLUSION

In the light of the above report, the following conclusions can be drawn:

1. Despite controversy, successful results are obtained in early treatment.
2. There is no best time of treatment; functional problems should be treated as soon as possible.
3. Treatment objectives must be firmly established; removal of etiologic factors and pressure habits and then correction of skeletal dysplasia.
4. Always inform patients about the possible di-phasic nature of treatment and even orthosurgical correction in adult complex anterior open bite deformity.
5. Further studies are recommended to evaluate relapse tendency and long-term stability of corrected anterior open bite in growing patients.

## Figures and Tables

**Fig. (1):**
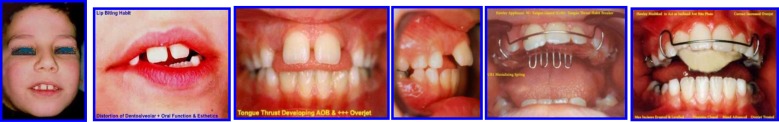
The sequence of interceptive (Phase I) Treatment: Hawley Removable Appliance with Habit Breaker & Tongue Crib to restore normal tongue position and function. Six months later, an anterior acrylic inclined plane has been constructed to guide the lower anterior teeth into normal overbite and Overjet. case # 1.
